# Impact of comorbidities on D-dimer and CRP in covid-19 patients: A comparative retrospective analysis

**DOI:** 10.6026/973206300220960

**Published:** 2026-02-28

**Authors:** Jyoti Shukla, Ritesh Yadav, Girish Dubey, Pankaj Sharma, Nishant Singh Verma

**Affiliations:** 1 Department of Biochemistry, Shrimant Rajmata Vijayaraje Scindia Medical College, Shivpuri, Madhya Pradesh, India; 2 Department of General Medicine, Shrimant Rajmata Vijayaraje Scindia Medical College, Shivpuri, Madhya Pradesh, India; 3 Department of Orthopaedics, Shrimant Rajmata Vijayaraje Scindia Medical College, Shivpuri, Madhya Pradesh, India

**Keywords:** COVID-19, D-dimer, C-reactive protein (CRP), comorbidities, inflammatory markers

## Abstract

The coronavirus disease 2019 (COVID-19) pandemic has posed significant challenges to healthcare systems, with inflammatory biomarkers emerging as important prognostic indicators. Therefore, it is of interest to evaluate the correlation between comorbidity burden and inflammatory markers-C-reactive protein (CRP) and D-dimer-in 100 hospitalized COVID-19 patients at a tertiary care center in Madhya Pradesh, India. Patients were categorized as non-comorbid (n=31), comorbid (n=30) and multimorbid (n=39), with a mean age of 53.18 ± 15.48 years and male predominance (63%). D-dimer and CRP levels were significantly higher in multimorbid patients compared to comorbid and non-comorbid groups (p<0.05). Thus, we show the importance of monitoring inflammatory biomarkers in COVID-19 patients with comorbidities for early identification of disease progression and targeted therapeutic intervention.

## Background:

The discovery of severe acute respiratory syndrome coronavirus 2 (SARS-CoV-2) at the end of 2019 led to the start of an unprecedented global pandemic that has radically changed the system of delivering health care to people all over the world [1]. Officially, this new pathogen was registered by the World Health Organization on December 31, 2019, in the presence of an atypical viral pneumonia outbreak, the center of which was Wuhan, Hubei, China [2]. The virus mostly invades lower respiratory tract and any other tissue containing angiotensin-converting enzyme 2 (ACE2) receptors causing a range of clinical outcomes such as asymptomatic infection to severe respiratory failure [3]. The pathophysiology of severe COVID-19 is the interaction of complex viral replication and immune responses of the host. Abnormal cytokine release also known as the cytotoxic storm is one of the critical factors of morbidity and mortality [4]. Such hyperinflammatory condition is marked by a significant increase in the level of pro-inflammatory mediators such as interleukin-6 (IL-6), tumor necrosis factor-alpha (TNF- 0) and interleukin-1 beta (IL-1 0), leading to tissue damage, acute respiratory disease syndrome (ARDS) and dysfunction of multiple organs [5]. Patients who have underlying comorbidities show increased susceptibility to life-threatening COVID-19. Ailments like high blood pressure, diabetes mellitus, kidney disease in chronic form and heart diseases have always been linked to a higher rate of hospitalization, intensive care unit admission and death [6]. The mechanisms involved include impaired immune, chronic low-grade inflammation and physiological reserve, which are together predisposing factors to dysregulated inflammatory responses in acute infection [7]. C-reactive protein (CRP) is an established acute-phase reactant that is mainly produced by hepatocytes through the stimulation of IL-6 [8]. It is a pentameric protein that is crucial in the innate immunity by complement activation, augmenting phagocytosis.

CRP levels in serum have been widely used as inflammatory biomarkers in COVID-19 and high levels of the serum have been associated with disease severity and poor prognosis [9]. Nevertheless, the difference in CRP in patients with multiple comorbidities is not sufficiently described. D-dimer is a fibrin degradation product, which is a sensitive biomarker of thrombotic disorders and coagulation activation [10]. Normal values are usually below 0.5 mg/L but it also can rise with age, pregnancy and other pathological conditions. High D-dimer concentration is indicative of the exaggerated coagulopathy of severe infection in COVID-19 and the level of D-dimer on admission has been shown to be useful in predicting the severity of disease and mortality [11]. The COVID-19-related coagulopathy is characterized by endothelial dysfunction, platelet activation and fibrinolytic dysfunctions leading to venous and arterial thrombotic complications [12]. Past studies have indicated that there are strong associations between COVID-19 outcomes and inflammatory biomarkers. Research has stated that a CRP of more than 100 mg/L indicates critical illness, whereas a dopimer of more than 1000 ng/mL indicates a higher risk of mortality [13]. Moreover, studies have pointed out that patients with diabetes mellitus have exaggerated inflammatory reactions with tremendously higher levels of CRP than non-diabetic people [14]. Also, chronic kidney disease has been linked with an increased baseline inflammation that can be increased in case of an acute infection [15]. Although there is growing evidence on individual biomarkers and individual comorbidities, there is scanty research effort that was done systematically to understand the cumulative effect of much comorbidity on COVID-19 patients who have inflammatory markers. The clinical significance of these relationships is that they have a significant role in risk and prognostication as well as in making therapeutic decisions. Multimorbidity as the co- occurrence of two or more chronic conditions is a concept that raises specific issues in the management of infectious diseases, which are worth specific research [16]. Therefore, it is of interest to assess the connection between the burden of comorbidity and the inflammatory (CRP and D-dimer) indicators in COVID-19 patients.

## Materials and Methods:

## Study design and setting:

This retrospective observational cohort study was carried out in one of the tertiary care teaching hospitals that were associated with a medical college in the town of Shivpuri, Madhya Pradesh, India. The institution is a referral center in the region with wide- ranging medical services offered to a wide range of patients. The data of the research were obtained at the medical records department after obtaining permission of the Institutional Health Research Ethics Committee. The research followed the guidelines in the Declaration of Helsinki and patient confidentiality was achieved during the research by removing irrelevant variables in the study and extracting only the variables that were relevant but that did not involve the personal details of the patients.

## Duration of the study and population:

The research was conducted over a six months period between the months of April and September 2025. Patients who had laboratory- confirmed COVID-19 infection were assessed to be eligible based on medical records of patients admitted with COVID-19 infection.

## Inclusion criteria:

Inclusion criteria were as follows: (1) confirmed COVID-19 diagnosis through reverse transcriptase-polymerase chain reaction (RT-PCR) test; (2) recorded CRP and D-dimer levels in the hospital; (3) detailed clinical records with demographic data, comorbidity (status) and associated laboratory parameters; (4) age eighteen and above at the time of hospital admission.

## Exclusion criteria:

The exclusion criteria included the following: (1) missing medical records, which did not contain important study variables; (2) pregnancy; (3) active malignancy; (4) chronic liver disease; (5) known coagulation disorders; (6) having other acute infections other than the COVID-19 infection; (7) taking anticoagulation therapy before admission.

## Sample size and categorization of patients:

One hundred and twenty-four records of COVID-19 patients were first screened to be eligible. Twelve records were eliminated because they did not meet the inclusion criteria or had some exclusion criteria making the final analysis sample 100 patients. The participants were divided into three different categories of participants according to the status of comorbidity: non-comorbid (no known chronic conditions), comorbid (only one chronic condition) and multimorbid (two or more chronic conditions). The comorbidities measured were hypertension, diabetes mellitus, chronic kidney disease, ischemic heart disease and chronic respiratory diseases.

## Data collection:

Individual medical records were scanned to obtain the relevant data on a data collection form. The variables gathered were demographic (age, gender), comorbidity and certain conditions, laboratory parameters (CRP, D-dimer, complete blood count, tests of renal functioning) and clinical outcomes. Immunoturbidimetric assay was used to measure CPR and the values were reported in milligrams per liter (mg/L). The amount of D-dimer was measured with the help of the latex-enhanced immunoassay and the results were presented in milligrams per liter (mg/L). Measures were taken in all laboratory measurements in the central laboratory of the hospital according to the standard operating procedures with proper quality control.

## Statistical analysis:

Statistical analysis and data entry were done through Statistical Package of Social Sciences (SPSS) ver. 27.0 (IBM Corporation, Armonk, NY, USA). Calculations of descriptive statistics were performed in the form of frequencies and percentages of categorical variables and means with standard deviations of continuous variables. The Shapiro-Wilk test was used to test normality of continuous variables. One way analysis of variance (ANOVA) was used when comparing (normally distributed) variables, whereas the Kruskal-Wallis test was used when comparing (non-normally distributed) variables. Appropriate pairwise comparisons were done after hoc using the honestly significant difference test of Tukey or Dunn. Two-group comparisons were done using independent samples t-test or Mann-Whitney U test. The pearson correlation coefficients were estimated to compare the relationship between continuous variables. All analyses were taken to be significant when it had a p-value that is less than 0.05.

## Results:

The study population comprised 100 patients with confirmed COVID-19 infection. The mean age was 53.18±15.48 years, ranging from 26 to 93 years. Males constituted the majority of participants (63%), while females represented 37% of the sample. Age distribution analysis revealed that 45% of patients were younger than 50 years, with the remaining 55% aged 50 years or older. Regarding comorbidity classification, 31 patients (31%) were categorized as non-comorbid, 30 patients (30%) as comorbid with a single chronic condition and 39 patients (39%) as multimorbid with two or more chronic conditions. The demographic characteristics across study groups are presented in Table 1.

The multimorbid group demonstrated significantly older age compared to other groups (p<0.001). Among patients with comorbidities, hypertension was the most prevalent condition (52 patients), followed by diabetes mellitus (48 patients), ischemic heart disease (18 patients) and chronic kidney disease (12 patients). Descriptive statistics for CRP and D-dimer levels across the entire study population revealed considerable variability. The overall mean CRP level was 94.95±105.24 mg/L, with values ranging from 5.5 to 600 mg/L. The overall mean D-dimer level was 5.07±5.9 mg/L, ranging from 0.2 to 20 mg/L. The distribution of biomarker levels across comorbidity groups is presented in Table 2. Analysis revealed a clear gradient in inflammatory biomarker concentrations across comorbidity categories. D-dimer levels demonstrated a nearly four-fold increase from non-comorbid to multimorbid patients. Similarly, CRP levels showed a progressive elevation with increasing comorbidity burden, with multimorbid patients exhibiting values approximately 2.5 times higher than non-comorbid individuals. Further analysis examined the relationship between specific comorbidities and biomarker levels. Patients with diabetes mellitus demonstrated significantly elevated CRP levels (112.34±89.45 mg/L) compared to non-diabetic patients (74.23±98.67 mg/L, p=0.028). Those with hypertension showed higher D-dimer levels (6.12±5.89 mg/L) compared to normotensive patients (3.45±4.23 mg/L, p=0.012). The detailed analysis of biomarker levels stratified by specific comorbidities is presented in Table 3. Chronic kidney disease showed the strongest association with elevated biomarkers, with affected patients demonstrating D-dimer levels more than double those of patients without renal impairment. Correlation analysis revealed positive associations between age and both D-dimer (r=0.42, p<0.001) and CRP (r=0.38, p<0.001) levels. No significant differences in biomarker levels were observed between male and female patients.

## Discussion:

The current research study can be useful in terms of attempting to understand the correlation between the comorbidity burden and the inflammatory biomarker profiles in patients with COVID-19. The results of our study indicate a distinct correlational relationship between the growing comorbidity status and the rise of both CRP and D-dimer, which proves the hypothesis that comorbidity conditions exacerbate the inflammatory response in the context of an SARS-CoV-2 infection. The incremental rise of the inflammatory markers among comorbidity groups found in the present study is consistent with the existing knowledge on the COVID-19 pathophysiology. Past studies have shown that patients who are already ill have an increased vulnerability to extremities of the disease [17]. High inflammatory reaction is probably due to default immune regulation found in chronic conditions which is worsened during acute infection with a virus. The average level of D-dimer of 8.27 + 7.07 mg/L of the multimorbid patients is significantly higher than the threshold levels that were considered to be associated with an adverse outcome. It has been determined that D-dimer levels more than 1000 ng/mL are predictive of an elevated risk of mortality in Covid-19 [18].

The significantly high concentrations of our multimorbid group indicate a strong activation of the coagulatory system, which is typical of the COVID-19-related coagulopathy, which is characterized by endothelial damage, dysfunctional platelets and fibrinolytic disbalances [19]. The CRP results indicate that multimorbid patients have the mean values of 137.05 mg/L of the CRP, as opposed to the non-comorbid patients with a mean of 53.58mg of the same. These findings are in line with the reported past observations, that the level of CRP is linked to the severity of the disease in COVID-19 20]. The much increased CRP levels on patients with multiple comorbidities indicate the accumulation of the inflammatory load on chronic conditions that added to acute infection. Specific comorbidity analysis showed that chronic kidney disease showed the best correlation with the elevation of biomarkers. D-dimer levels were about two times higher in patients with renal impairment as compared to patients with normal renal function. This observation is supported by the fact that there is an inflammatory condition known to be present in uremia and the fact that there is a diminished removal of acute-phase reactants in chronic kidney disease [21]. Moreover, kidney disease is one of the causes of endothelial injury and coagulation that can increase thrombotic complications caused by COVID-19. There were strong correlations between diabetes mellitus and high CRP levels, which confirms earlier studies indicating that diabetic COVID-19 patients have exaggerated inflammatory responses [22]. Hyperglycemia facilitates oxidative stress, immune protein glycation and neutrophil dysfunction, all of which contribute to increased production of cytokines in case of infection. The low-grade inflammation that is chronic and typical of diabetes offers a substrate to which acute inflammation caused by the infection is superimposed. Hypertension was also linked with the increased D-dimer, which may be the evidence of the endothelial dysfunction that is the part of the chronic hypertensive conditions. Earlier studies have found hypertension as a risk factor on its own to cause severe effects of COVID-19 outcomes [23]. The elevations of the biomarkers could be caused by the dysregulation of the renin-angiotensin system linked to hypertension and infection with SARS-CoV-2. The correlatively positive relationship between age and the levels of inflammatory biomarkers that were found in this paper corresponds to the idea of immunosenescence and inflammaging [24]. Increased inflammatory markers and impaired regulatory ability in the immune system with age may be linked to predisposing older people to more severe inflammatory reactions when they become acutely infected. Age distributions in our comorbidity categories, where older patients were in the multimorbid category, might have served as the cause of biomarker patterns.

Lack of any significant gender differences in the levels of biomarkers in our research is contradicted by some of the past studies that indicated that males dominate severe COVID-19 [25]. Nonetheless, the sample size is relatively small and it may not be enough to yield statistically significant results of gender-specific differences. Moreover, the presence of hormonal and genetic effects on immune responses can interact with comorbidity status in complex ways in order to change the inflammatory markers. These clinical implications of findings are enormous. The results of the analysis of markedly increased levels of inflammatory indicators in multimorbid COVID-19 patients contribute to implementing increased monitoring measures in relation to this risk group. Early detection of the disease at risk, using the biomarker analysis, could result in a timely therapeutic intervention, such as anticoagulation, immunomodulatory therapy and intensive supportive care [26]. The findings of the study also reflect the necessity to focus on determining cumulative comorbidity burden instead of single diseases and conditions when examining the risk of COVID-19. Multimorbidity is a concept that involves complicated interaction between chronic diseases, which can get synergies in relation to inflammatory reactions. The use of clinical risk stratification instruments that use comorbidity counts along with biomarker values could enhance prognostic accuracy [27]. Regular assessment of CRP, D-dimer, and ferritin levels in patients with COVID-19 can aid in early risk stratification and prompt therapeutic decision-making, thereby potentially enhancing clinical outcomes and reducing mortality [28]. There are a number of weaknesses to this study that should be mentioned. The retrospective design does not permit the causation of relationships and is prone to selection and information bias due to medical record review. The single-center environment can be a weakness, as it might not be applicable to other populations with varying demographic and clinical profiles. The small sample size, which was sufficient to identify relevant differences among groups, did not allow elaborate subgroup analysis of particular forms of comorbidity. Also, serial biomarker measurements were not being done and this did not allow to measure dynamic changes throughout the disease course. The next line of research efforts should be prospective multicenter studies with bigger sample size to confirm these results and investigate the prognostic value of biomarker-comorbidity interactions. The supplementary inflammatory markers such as IL-6, ferritin and procalcitonin could be investigated to complete the information. Research that determines the effect of comorbidity management optimization on inflammatory reactions during COVID-19 may guide preventive measures.

## Conclusion:

Comorbidities play a crucial role in influencing disease severity and clinical outcomes in COVID-19 patients. Elevated inflammatory markers reflect increased risk in patients with multiple underlying conditions. Early identification and careful monitoring of high-risk individuals can support timely intervention and improved patient management.

## Figures and Tables

**Table 1:** Demographic characteristics of study population by comorbidity group

**Variable**	**Non-comorbid (n=31)**	**Comorbid (n=30)**	**Multimorbid (n=39)**	**Total (n=100)**	**p-value**
**Age (years)**					
Mean ± SD	42.35 ± 12.67	54.23 ± 13.45	61.18 ± 14.21	53.18 ± 15.48	<0.001
Range	26-68	32-78	35-93	26-93	
**Age Groups**					
<50 years	22 (70.97%)	13 (43.33%)	10 (25.64%)	45 (45%)	<0.001
≥50 years	9 (29.03%)	17 (56.67%)	29 (74.36%)	55 (55%)	
**Gender**					
Male	21 (67.74%)	18 (60.00%)	24 (61.54%)	63 (63%)	0.782
Female	10 (32.26%)	12 (40.00%)	15 (38.46%)	37 (37%)	

**Table 2:** D-Dimer and CRP levels according to comorbidity groups

**Variable**	**Statistic**	**Non-comorbid (n=31)**	**Comorbid (n=30)**	**Multimorbid (n=39)**	**p-value**
**D-Dimer (mg/L)**					
	Mean ± SD	2.21 ± 2.89	3.80 ± 4.01	8.27 ± 7.07	<0.001
	Median	0.7	2.62	6.5	
	Minimum	0.2	0.43	0.8	
	Maximum	16	17.89	20	
**CRP (mg/L)**					
	Mean ± SD	53.58 ± 108.45	71.59 ± 56.32	137.05 ± 98.60	<0.001
	Median	29	61	112	
	Minimum	5.5	6.4	12.8	
	Maximum	600	201	449	

**Table 3:** Biomarker levels stratified by specific comorbidities

**Comorbidity**	**n**	**D-Dimer (mg/L) Mean ± SD**	**p-value**	**CRP (mg/L) Mean ± SD**	**p-value**
**Hypertension**					
Present	52	6.12 ± 5.89	0.012	105.67 ± 95.34	0.034
Absent	48	3.45 ± 4.23		78.45 ± 102.56	
**Diabetes Mellitus**					
Present	48	5.89 ± 6.12	0.045	112.34 ± 89.45	0.028
Absent	52	4.02 ± 5.34		74.23 ± 98.67	
**Chronic Kidney Disease**					
Present	12	9.45 ± 6.78	0.008	156.78 ± 112.34	0.006
Absent	88	4.34 ± 5.23		83.56 ± 89.45	
**Ischemic Heart Disease**					
Present	18	7.23 ± 5.67	0.023	128.45 ± 78.90	0.018
Absent	82	4.45 ± 5.78		86.34 ± 102.34	
**Gender**					
Male	63	5.34 ± 6.12	0.456	98.67 ± 108.45	0.523
Female	37	4.67 ± 5.45		89.23 ± 98.76	
